# Experimental evaluation of laparoscopic laser speckle contrast imaging to visualize perfusion deficits during intestinal surgery

**DOI:** 10.1007/s00464-022-09536-9

**Published:** 2022-09-06

**Authors:** Wido Heeman, Aurelia C. L. Wildeboer, Mahdi Al-Taher, Joost E. M. Calon, Laurents P. S. Stassen, Michele Diana, Joep P. M. Derikx, Gooitzen M. van Dam, E. Christiaan Boerma, Nicole D. Bouvy

**Affiliations:** 1 Faculty Campus Fryslân, University of Groningen, Leeuwarden, 8911 CE The Netherlands; 2 Department of Surgery, Optical Molecular Imaging Groningen, University Medical Centre Groningen, Groningen, 9713 GZ The Netherlands; 3 LIMIS Development BV, Leeuwarden, 8934 AD The Netherlands; 4 Department of Pediatric Surgery, Amsterdam UMC, 1105 AZ Amsterdam UMC, The Netherlands; 5 GROW, School for Oncology and Developmental Biology, Maastricht University, Maastricht, 6229 ER The Netherlands; 6 IRCAD, Research Institute Againstgainst Digestive Cancer, 67000 Strasbourg, France; 7 Department of Surgery, Maastricht University Medical Center, Maastricht, 6200 MD The Netherlands; 8 ZiuZ Visual Intelligence BV, Gorredijk, 8401 DK The Netherlands; 9 NUTRIM School of Nutrition and Translational Research in Metabolism, Maastricht University Medical Center, Maastricht, 6229 HX The Netherlands; 10 Medical Centre Leeuwarden, Department of Intensive Care, Leeuwarden, 8934 AD The Netherlands

**Keywords:** Intestinal persfusion, Anastomosis, Ischemia, Perfusion assessment, Anastomotic leakage, Bowel viability

## Abstract

**Background:**

Ischemia at the site of an intestinal anastomosis is one of the most important risk factors for anastomotic leakage (AL). Consequently, adequate intestinal microperfusion is essential for optimal tissue oxygenation and anastomotic healing. As visual inspection of tissue viability does not guarantee an adequate objective evaluation of intestinal microperfusion, surgeons are in dire need of supportive tools to decrease anastomotic leakage after colorectal surgery.

**Methods:**

In this feasibility study, laparoscopic laser speckle contrast imaging (LSCI) was used to evaluate intestinal microperfusion in an experimental ischemic bowel loop model. Both large and small ischemic loops were created from the small intestine of a pig; each loop was divided into 5 regions of interest (ROI) with varying levels of ischemia. Speckle contrast and local capillary lactate (LCL) was measured in all ROIs.

**Results:**

Both real-time visualization of intestinal microperfusion and induced perfusion deficits was achieved in all bowel loops. As a result, the emergence of regions of intestinal ischemia could be predicted directly after iatrogenic perfusion limitation, whereas without LSCI signs of decreased intestinal viability could only be seen after 30 minutes. Additionally, a significant relation was found between LCL and LSCI.

**Conclusion:**

In conclusion, LSCI can achieve real-time intraoperative visualization of intestinal microperfusion deficits, allowing for accurate prediction of long-term postoperative ischemic complications. With this revealing capacity, LSCI could potentially facilitate surgical decision-making when constructing intestinal anastomoses in order to mitigate ischemia-related complications such as AL.

Anastomotic leakage (AL) remains one of the most frequently occurring and feared complications in gastrointestinal surgery. In AL, the enteric content contaminates the peritoneal cavity and causes high morbidity and mortality rates. Despite evolving surgical techniques and improved perioperative care, the average reported incidence of AL is 10%, which has not significantly declined over the last decades ‘[1–4]. Although the exact etiology of AL remains a difficult conundrum, adequate anastomotic perfusion has been identified as one of the most important prerequisites for optimal anastomotic healing [5]. When inadequate anastomotic perfusion is left unaltered, an improvement in anastomotic perfusion from increased collateral circulation is unlikely to develop within the first five postoperative days [6], compromising anastomotic regeneration. Having an accurate indication of the local intestinal perfusion quality can help the surgeon to identify the optimal anastomotic site in order to prevent AL. As a result, intraoperative assessment of bowel perfusion is a vital element in surgical decision-making when performing restorative intestinal resections [6]. However, the traditional direct visual inspection of the intestinal tissue for signs of viability (e.g., mucosal color, pulsatile bleeding from marginal arteries) is highly subjective and does not allow for an adequate evaluation of intestinal microperfusion [6–8]. For this reason, numerous techniques, such as near-infrared fluorescence angiography and the assessment of various parameters of intestinal viability have recently been developed. Unfortunately, due to a lack of convincing evidence for the use of these existing techniques, there is still no general consensus regarding their clinical impact [6], leaving surgeons with a dire need for alternative tools. Horgan and Gorey defined 5 criteria for an ideal bowel viability test, namely (1) ready-to-use availability in every operating room, (2) the technique should require no other specialist personnel other than the surgeon, (3) maximum accuracy with a minimum of false-negative and, more importantly, few false positive results, (4) objectivity and reproducibility, and (5) cost-effectiveness (6).

We hypothesize that laser speckle contrast imaging (LSCI) can give an accurate indication of local intestinal perfusion quality. LSCI is a dye-free, non-contact, non-invasive, and full-field perfusion imaging technique which can deliver 2D perfusion images in real time [9]. The technique uses laser light to generate a so-called speckle pattern which changes due to motion of scatterers, or red blood cells in this case. In this study, we tested the feasibility and potential of laparoscopic LSCI for real-time intraoperative visualization of intestinal perfusion.

## Materials and methods

This study was performed at the central animal facility of Maastricht University (Maastricht, The Netherlands). The animal was handled in compliance with the regulations of the Dutch legislation for animal research and the ARRIVE guidelines [10] following a protocol approved by the Experimental Animal Committee of Maastricht University (DEC-UM) under working protocol number 2017-021-001.

### Animal experiment

One female Dutch landrace pig, weighing 35.5 kg, was used for the current experiment. Following an acclimatization period in the animal keeping facility, an intramuscular injection of Zolazepam/Tiletamine 6 mg/kg (Virbac, Barneveld, The Netherlands) and Thiopental 10 mg/kg (Panpharma SA, Trittau, Germany) was given as premedication. Anesthesia was induced through an intravenous injection of Sufentanyl 0.01 mg/kg/h (Hameln Pharma GmbH, Hameln, Germany), Propofol 9 mg/kg/h (B. Braun Melsungen AG, Melsungen, Germany), and Midazolam 1 mg/kg/h (Aurobindo, Baarn, The Netherlands). After intubation, the pig was continuously mechanically ventilated and anesthesia was deepened with an additional administration of Sufentanyl and Propofol when deemed necessary. At the end of the procedure, the animal was sacrificed with a lethal dose of Pentobarbital 200 mg/kg (AST Farma, Oudewater, The Netherlands).

### Surgical procedure

**Fig. 1:**
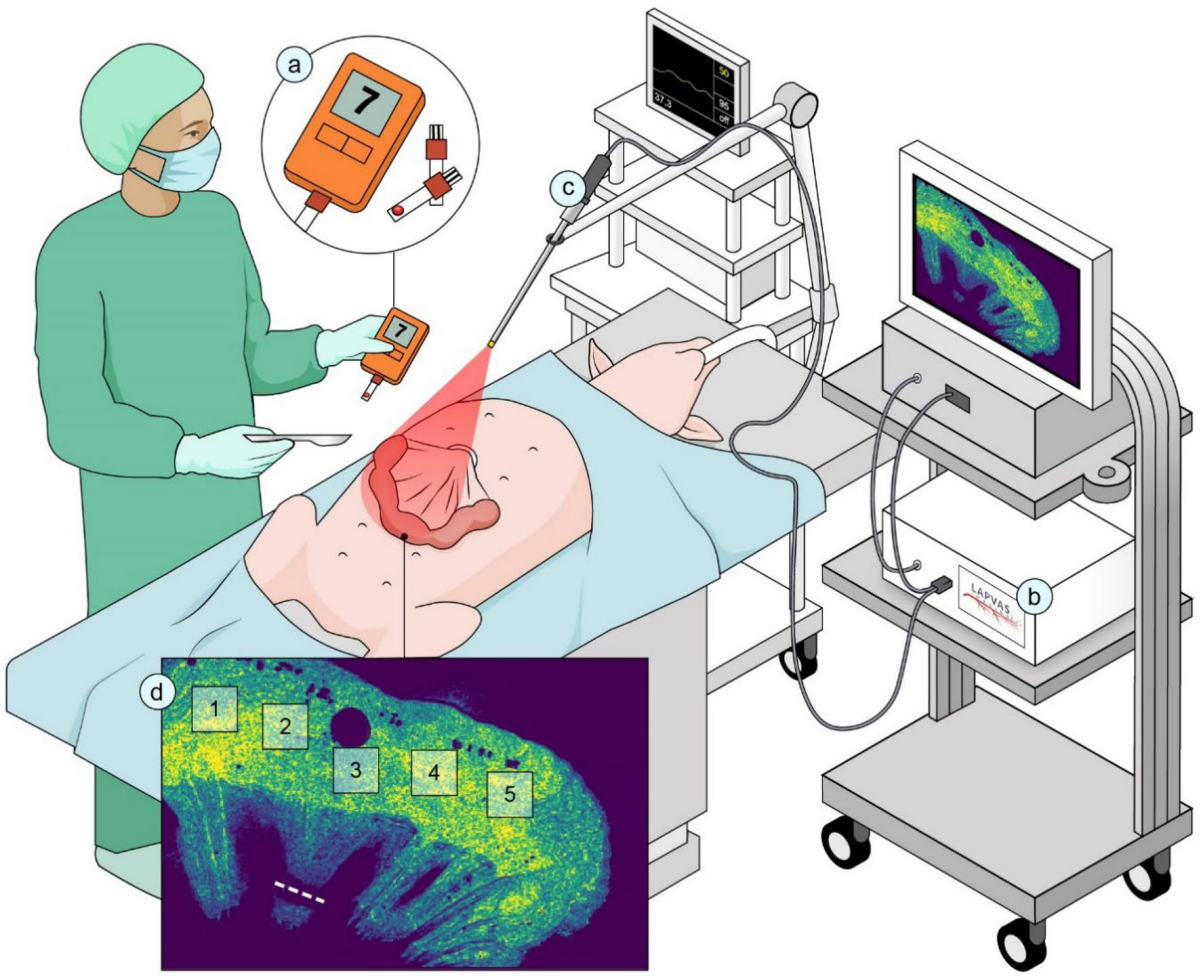
Graphic representation of the experimental setup. (a) Local capillary lactate analyzer, (b) the PerfusiX-Imaging laparoscopic laser speckle contrast imaging setup, (c) mounted laparoscope set at 15 cm above the intestine, and (d) marked positions of the regions of interest on the bowel serosa together with a dotted line indicating the vascular clipping site at the base of the central ischemic region (ROI 3). *Illustration made by* *Sieben Medical Art, *© 2022 Sieben Medical Art

Access to the peritoneum and the small bowel was obtained through a midline laparotomy. Four intestinal loops, with a length of approximately 15 cm, were randomly selected. At the mesenteric side of the first two loops, tissue perfusion was compromised by clipping ~ 7 peripheral arteries and veins using a vessel-clipping device (Endo Clip 10 mm Pistol Grip single use clip applier, Medtronic, Dublin, Ireland). To investigate the ability of LSCI to distinguish very subtle changes in perfusion, an additional set of two smaller loops was created. In these loops, tissue perfusion was compromised by the clipping of ~ 4 peripheral arteries and veins. In each bowel loop, 5 regions of interest (ROIs) were identified and marked with a surgical marker pen (Fig. 1). The regions were located as follows: (1 and 5) the two outer regions, located at the lateral borders of the loops, and (2 and 4) the two inner regions adjacent to 1 and 5, flanking the central region of the ischemic loop (3). This ischemic bowel loop model was a simulation of a model which was previously described [11, 12]. Images were recorded right before perfusion limitation (baseline), right after perfusion limitation (*T* = 0), and after 30 (*T* = 30) or 45 min (*T* = 45), respectively.

### Local capillary lactate levels

Local capillary lactate (LCL) levels were measured in chosen ROI by puncturing bowel serosa with a 23 Gauge needle (BD Switzerland Sarl, Eysins, Switzerland) and analyzing this blood with an EDGE lactate analyzer (ApexBio, Taipei, Taiwan, People’s Republic of China). LCL levels were obtained during all intestinal perfusion assessments.

### Laparoscopic laser speckle imaging set-up and data acquisition

PerfusiX-Imaging (LIMIS Development BV, Leeuwarden, The Netherlands) device was used to acquire laparoscopic LSCI images [13]. PerfusiX-Imaging is a laparoscopic perfusion imager which works with standard laparoscopic equipment, namely an Olympus laparoscopic video system (OTV-S200, Olympus, Hamburg, Germany) and a 30-degree laparoscope (EndoEye, Olympus, Hamburg, Germany) in this case. It provides real-time 2D-perfusion maps instantaneously and continuously. LSCI requires a laser light source to illuminate the tissue of interest. The random interference pattern then forms an interference pattern on the camera sensor, i.e., the so-called speckle pattern. This pattern changes with movement of underlying red blood cells, in a rate which corresponds to the amount of blood flow. Hence, it is this blurring or loss in contrast which is quantified as laser speckle perfusion units (LSPU) [9]. Higher LSPU values correspond to better tissue perfusion compared to lower LSPU values. The LSPU are defined as the ratio of standard deviation divided by the mean intensity (Eq. 1).

1 $$LSPU=\frac{\sigma }{<I>}$$

LSCI is a fast and full-field imaging technique which can image large surfaces without the need for a contrast agent. Non-invasive subsurface perfusion measurements are characterized by means of high spatial and temporal resolution. The device houses a 639 nm laser and allows for a fast, instant switching between conventional white light and laser light. The laser was connected to the laparoscope using the unmodified optical fiber attached to the EndoEye (Fig. 1). The 2D perfusion maps were directly available in the operating room. The laparoscope was mounted onto a frame directly above the operating table at a fixed distance of 15 cm from the operating field to standardize measurement conditions and create a static optical fiber [14] (Fig. 1). The camera exposure time was 20 ms for all measurements. The aperture could not be determined. However, it was kept constant with a speckle/pixel ratio larger than one, satisfying the Nyquist criterion [15]. Images were acquired at 50 frames per second. The images were analyzed using the PerfusiX-Imaging software suite (LIMIS Development BV, Leeuwarden, The Netherlands). Images were analyzed using spatial LSCI with a spatial window size of $7 x 7$ pixels.

On each loop, 5 ROI were marked corresponding to the vascularized (ROI 1&5), presumed marginally viable (ROI 2&4) and central ischemic (ROI 3, directly above clipped vessels) regions, respectively (Fig. 1D). LCLs were obtained by puncturing the bowel serosa in the same ROIs. The ROI size was ~ 1.5 × 1.5 cm. The average LSPU values of the ROIs of 25 frames were calculated and reported using Eq. 1.

### Statistical analysis

Basic statistics were used to analyze the results. A logarithmic curve estimation was performed to test the relation between LSPU values and LCL levels.

## Results

### Animal experiment and surgical procedure

The surgical procedure was performed without any complications or adverse events.

### Laser speckle perfusion assessment

**Fig. 2:**
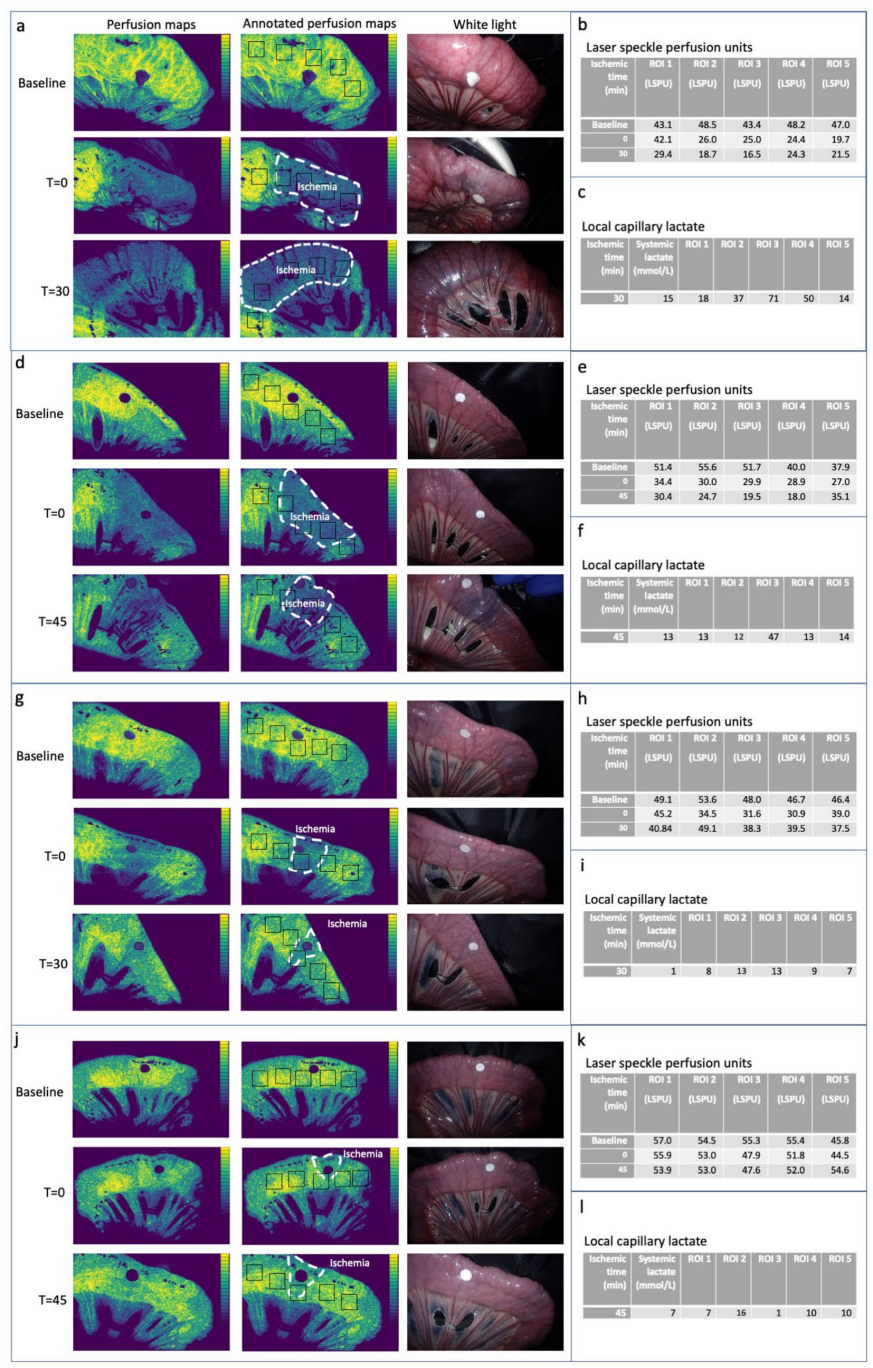
Overview of all 2D perfusion maps, corresponding white light images and the LSPU and LCL data captured from all ROIs. **a** Representative perfusion maps of the large ischemic bowel loop and annotated perfusion maps with ischemia indication and the location of the ROI. And corresponding white light images at baseline, 0, and 30 min of iatrogenic perfusion limitation. **b** Laser speckle perfusion units (LSPU) calculated for corresponding ROIs. **c** LCL levels in the ROIs located in a large ischemic bowel loop after 30 min of iatrogenic perfusion limitation. **d** Representative perfusion maps of a large ischemic bowel loop and annotated perfusion maps with ischemia indication and the location of the ROI. And corresponding white light images at baseline, 0, and 45 min of iatrogenic perfusion limitation. **e** LSPU calculated for corresponding ROIs. **f** LCL levels in the ROIs located in a large ischemic bowel loop after 45 min of iatrogenic perfusion limitation. **g** Representative perfusion maps of a small ischemic bowel loop and annotated perfusion maps with ischemia indication and the location of the ROI. And corresponding white light images at baseline, 0, and 30 min of iatrogenic perfusion limitation. **h** LSPU calculated for corresponding ROIs. **i** LCL levels in the ROIs located in a small ischemic bowel loop after 30 min of iatrogenic perfusion limitation. **j** Representative perfusion maps of a small ischemic bowel loop and annotated perfusion maps with ischemia indication and the location of the ROI. And corresponding white light images at baseline, 0, and 45 min of iatrogenic perfusion limitation. **k** LSPU calculated for corresponding ROIs. **l** LCL levels in the ROIs located in a small ischemic bowel loop after 45 min of iatrogenic perfusion limitation

Real-time visualization of intestinal microperfusion was achieved using PerfusiX-imaging in all bowel loops resulting in analyzable 2D speckle contrast images. The 2D perfusion maps allowed us to make a clear distinction between adequately and poorly perfused tissue regions in both large (Fig. 2a and 2d) and small ischemic loops (Fig. 2g and 2j). For larger loops, LSPU values (Fig. 2b and 2e) and corresponding perfusion maps of *T* = 0 seemed to predict the emergence of ischemic tissue regions, which could only be seen in white light at *T* = 30/*T* = 45. In smaller loops (Fig. 2h and 2k), visualized perfusion deficits at *T* = 0 were much larger than those later observed at *T* = 30/*T* = 45.

### Local capillary lactate levels

LCL levels in all ROIs at the time of laser speckle perfusion assessment are presented in Fig. 2c and 2f (large loops) and Fig. 2i and 2l (small loops). LCL levels below 0.60 mmol/L were expressed as ‘low’ using the lactate analyzer. For the statistical analysis, all ‘low’ LCL levels were regarded as 1 mmol/L in order to standardize and prevent underestimation. When comparing LCL levels measured in each of the ROIs within each bowel loop, no significant differences in LCL levels were found for smaller loops. However, LCL levels for larger loops did show a steep increase in LCL. The logarithmic curve estimation of the relationship between measured LSPU and LCL levels revealed a significant relation (*p* = 0.013) with an *R*-value of 0.55, indicating a moderate correlation.

## Discussion

In this animal study, we successfully acquired laser speckle contrast images during intestinal surgery using the PerfusiX-Imaging laparoscopic LSCI setup. With this laparoscopic perfusion imager, we were able to detect both small and large intestinal perfusion deficits in real time. This was in contrast to visible light, which only allowed for the detection of compromised intestinal perfusion no sooner than 30 min.

Measurement of LCL levels was chosen as an objective reference for LSCI findings. LCL reflects tissue oxygenation status of intestinal cells, and its use is validated in both experimental and clinical perfusion imaging studies [11, 16, 17]. In this study we observed a clear correlation between LSPU values and lactate levels in larger ischemic loops. As anticipated, impaired perfusion was accompanied by a rise in lactate levels. However, this relation was less apparent in smaller ischemic loops, in which (relative) changes in LSPU values were not accompanied by changes in LCL. The overall logarithmic correlation of combined small and large areas could indicate that only below a critical level of perfusion, tissue becomes hypoxic, causing a steep rise in LCL. This phenomenon is commonly seen in other hemodynamic processes (e.g., hemodilution and venous-to-arterial carbon dioxide gradient related to dysoxygenation [18], i.e., an imbalance between oxygen supply and demand. Future studies into this cut-off value may support clinical decision-making to prevent irreversible tissue damage.

Observed characteristics of LSCI seem of particular interest during construction of intestinal anastomoses. In this setting, an adequate intraoperative assessment of intestinal microperfusion is required to ascertain the viability of the newly formed anastomosis to prevent ischemia-related complications such as AL. Traditional visual inspection by the surgeon has proven to be highly subjective and has little predictive value [7]. This fueled development of perfusion imaging techniques such as near-infrared fluorescence imaging, a promising technique which has gained a steep increase in popularity recent decade [19, 20]. However, a limitation of fluorescence angiography applications lies in the need for specific fluorescent dyes. Besides the fact that the injection of a dye carries certain risks (e.g., allergic reactions and toxicity), the use of dyes creates an opportunity for error in the execution and interpretation of perfusion assessment especially if repeated measurements are required. Indocyanine green (ICG) fluorescence angiography, for instance, requires the binding of ICG to blood proteins in order to visualize blood flow. When used incorrectly, ICG fluorescence angiography measures the binding of the fluorescent dye to blood proteins rather than the flow of red blood cells [21]. This could lead to a situation where the measured fluorescence intensity is caused by a difference in dye concentration rather than by a change in blood flow. Advantages of LSCI over fluorescence angiography include the ability to repetitively and continuously measurement of perfusion as there is no wash-out effect. In addition, LSCI is a full-field imaging modality and allows for real time and direct quantification of entire organ perfusion. Laparoscopic LSCI is still an uncommon perfusion imaging technique that requires further clinical validation. Next steps should include studies with regards to the ability to detect perfusion deficits in colorectal surgery and ultimately the effect of laparoscopic LSCI on anastomotic leakage rates.

Despite succeeding to detect intestinal perfusion deficits using the PerfusiX-imaging laparoscopic LSCI setup, some limitations regarding this study need to be addressed. Although we performed multiple measurements on 4 bowel loops, we only used one animal. Therefore, the data presented in this study should be treated with caution as they are limited to allow for solid conclusions. As our results contain no unreasonable outliers, we do expect to see similar results in a larger experiment. Moreover, to respect the directives on animal experimentation and the 3 R’s (Replacement, Reduction, Refinement) [22], the current set up was deemed sufficient to study the feasibility of this novel technique. The second limitation regards the choice of a small intestinal model. AL more frequently occurs in colorectal anastomoses than in small intestinal anastomoses [4], which merits a higher need for an adequate perfusion evaluation during surgery of the large intestine. Unfortunately, the spiral-like orientation of the porcine colon is not fitting for the creation of intestinal loops, hence the choice of our current model. Nevertheless, the porcine model does allow for the best translation of experimental results, as similarity to human intestinal physiology is high [23]. Another limitation is that throughout perfusion assessments in this study the distance between the camera and the bowel loops was kept constant to minimize data variations. Probably, in a clinical setting, this distance cannot be maintained at a fixed distance, which could potentially lead to altered perfusion assessment. Therefore, the system should be further studied in a minimally invasive setting, as this would be the clinical application. These studies should also focus on the normalization and quantification of the LSPU findings.

Altogether, the demonstrated feasibility of using laparoscopic LSCI to evaluate intestinal perfusion during surgery, together with the convincing results of this experimental study, trigger enthusiasm for a rapid clinical translation of this technology. However, the fine-tuning of the PerfusiX-Imaging technology, as well as additional experiments in both animal and human studies are required to further demonstrate the anticipated clinical usefulness.

## Conclusion

In conclusion, laparoscopic LSCI setup can achieve a real-time intraoperative visualization of intestinal perfusion deficits, allowing for accurate prediction of long-term postoperative ischemic complications. With this revealing capacity, LSCI could potentially facilitate surgical decision-making when constructing intestinal anastomoses in order to mitigate ischemia-related complications such as AL.
